# Mu-Rhythm Phase Modulates Cortical Reactivity to Subthreshold TMS: A TMS–EEG Study

**DOI:** 10.3390/bioengineering13040391

**Published:** 2026-03-27

**Authors:** Yuezhuo Zhao, Panli Chen, Wenshu Mai, Xin Wang, He Wang, Ying Li, Jiankang Wu, Zhipeng Liu, Jingna Jin, Tao Yin

**Affiliations:** 1 Institute of Biomedical Engineering, Chinese Academy of Medical Sciences and Peking Union Medical College, Tianjin 300192, China; yuezhuozhao200209@163.com (Y.Z.); panli_chen2022@163.com (P.C.); wenshumai@126.com (W.M.); wangxin121926@163.com (X.W.); whe19882006@126.com (H.W.); yinglibme@163.com (Y.L.); 13116127056@163.com (J.W.); lzpeng67@163.com (Z.L.); 2 Tianjin Key Laboratory of Neuromodulation and Neurorepair, Tianjin 300192, China

**Keywords:** phase-dependent responses, mu-rhythm, transcranial magnetic stimulation, electroencephalogram, subthreshold intensity

## Abstract

Background: The phase of electroencephalogram (EEG) signals critically influences cortical reactivity to external inputs. Phase-dependent effects and their sensitivity to stimulation intensity have been observed at suprathreshold levels, while subthreshold transcranial magnetic stimulation (TMS) cannot induce motor evoked potentials (MEPs), resulting in limited research on phase-dependent responses under subthreshold stimulation. In this study, we used a combined transcranial magnetic stimulation and electroencephalography (TMS–EEG) approach to examine how the ongoing EEG phase influences cortical responses at subthreshold intensity and to characterize these responses in terms of temporal, spatial, and spectral features. Methods: Thirty-four healthy adults received subthreshold single-pulse TMS at the motor hotspot during 64-channel EEG recording. The mu-phase at the time of TMS delivery was estimated using autoregression-based forward prediction and categorized into four bins (0°, 90°, 180°, and 270°). The cortical responses were assessed using inter-trial phase coherence (ITPC), TMS-evoked potentials (TEPs), global mean field power (GMFP), and event-related spectral perturbation (ERSP). Results: Phase estimation reliably distinguished four mu-phase bins. Subthreshold TMS–EEG responses showed clear phase dependence: early ITPC and several TEP components (N15, P30, N45, P60, and N100) differed significantly across phases, with 180° and 270° often eliciting stronger responses. GMFP revealed robust phase effects at mid-latency components, and TMS-induced mu-rhythms were the greatest at 180°. Conclusions: Our results showed that the EEG phase significantly modulates cortical reactivity at subthreshold stimulation levels, supporting mu-phase-based closed-loop TMS as a promising strategy for precise neuromodulation.

## 1. Introduction

Millisecond-scale fluctuations in ongoing neural electrical activity are a key factor contributing to trial-to-trial variability in cortical responses to external stimuli, a phenomenon that has been well-documented in the visual and sensorimotor cortices. For instance, in the visual cortex, the phase of local field potentials in the alpha band influences whether a visual stimulus is perceived [1,2]. Similarly, when transcranial magnetic stimulation (TMS) is delivered at different phases of the sensorimotor mu-rhythm, it evokes significantly different amplitudes of motor evoked potentials (MEPs) [3,4]. These findings suggest that, in addition to TMS parameters (such as intensity, coil position, and inter-stimulus interval), the electroencephalogram (EEG) phase at the moment of stimulation is an additional factor shaping cortical responses. Recently, the EEG phase-dependent closed-loop TMS method is developed, aiming to improve therapeutic efficacy and reduce response variability in TMS therapies.

In studies exploring the relationship between the EEG phase and TMS responses, MEPs are widely used to measure TMS-evoked responses due to their quantifiable and easily measurable characteristics. Zrenner et al. introduced an EEG phase-dependent TMS approach and reported that TMS applied during the trough of the sensorimotor mu-rhythm elicited higher MEP amplitudes compared to stimulation at the peak [5]. Both Wischnewski et al. and Schilberg et al. reported larger MEP amplitudes when the mu-rhythm was in the trough and rising phases, as demonstrated in real-time stimulation experiments and later summarized in a recent review [6,7,8]. Subsequently, Schaworonkow et al. not only replicated these findings but also revealed that the modulatory effect of the mu-phase on MEPs is more prominent at lower stimulation intensities [9]. Since these studies relied on MEP amplitudes to quantify cortical responses, they all employed suprathreshold stimulation intensities, thereby advancing our understanding of how EEG phase modulates cortical responses to suprathreshold TMS.

Subthreshold TMS is also widely used in neuromodulation therapies for stroke and Parkinson’s disease [10,11,12,13]. The cortical response to TMS is influenced by stimulation intensity [14,15]. As stimulation intensity increases, the population of activated neurons gradually expands, leading to a higher amplitude of the evoked response. Once the stimulation intensity increases to a certain level, the response plateaus and no longer increases [16]. Notably, phase-dependent modulation is more pronounced at intensities near the resting motor threshold (RMT) [17]. Taken together, these findings motivate the investigation of phase effects at subthreshold intensities. Desideri et al. reported phase-dependent modulation of subthreshold cortical responses at 90% RMT, with an enhanced N100 when TMS was delivered at the negative relative to the positive peak of the sensorimotor mu-rhythm [18]. However, their study was limited to a peak–trough comparison. The temporal, spatial, and spectral profile of subthreshold phase effects across finer phase bins therefore remains incompletely characterized.

Transcranial magnetic stimulation electroencephalography (TMS–EEG) enables direct assessment of cortical responses to TMS by recording TMS-evoked potentials (TEPs) and TMS-evoked oscillation. This approach provides a measure of intrinsic cortical excitability and large-scale network dynamics [19]. Unlike MEPs, TMS–EEG allows assessment of cortical reactivity independently of peripheral motor responses, making it suitable for probing subthreshold stimulation effects [20]. TMS–EEG features in the time, spatial, and time–frequency domains have been explored as biomarkers to characterize altered cortical excitability and network dynamics in neurological conditions and to support outcome prediction. For example, Tscherpel et al. reported that in stroke patients with similar clinical deficits but no elicitable MEPs, TMS–EEG revealed distinct cortical response patterns, making it suitable for assessing cortical responses beyond MEP-based measures [21].

This study employs TMS–EEG and an offline analysis framework to systematically and comprehensively investigate the influence of the EEG phase on cortical responses under subthreshold stimulation intensities. We focus on two questions: (1) whether the mu-rhythm phase influences cortical responses under subthreshold stimulation intensities and (2) whether there are significant differences in the time-domain, spatial, and time–frequency characteristics of the evoked responses across different mu-rhythm phases. Our study provides preliminary experimental support for understanding how mu-rhythm phases influence the cortical response to subthreshold TMS and might inform the development of phase-dependent closed-loop TMS methods.

## 2. Methods

### 2.1. Participants

Thirty-five healthy subjects (15 males, 20 females; mean age 23.6 ± 2.0 years) were recruited. After removing one subject because of severe EEG drift, the data from thirty-four subjects were included in the final analysis. All participants were right-handed as assessed by the Edinburgh Handedness Inventory [22]. All participants provided written informed consent in accordance with the Declaration of Helsinki. This study complied with the TMS safety guidelines of the International Federation of Clinical Neurophysiology [23,24] and was approved by the Ethics Committee of the Institute of Biomedical Engineering, Chinese Academy of Medical Sciences.

### 2.2. Procedures

**Figure 1:**
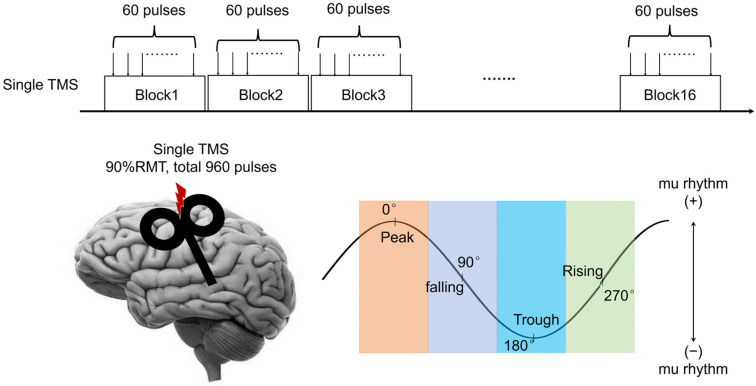
The depiction of the experimental procedure.

The subjects were instructed to remove all electronic devices from their body and clothing, to sit as still as possible, to keep their hands relaxed, and to maintain their gaze on a fixation cross throughout all measurement procedures. Resting and TMS–EEG data were recorded with a 64-channel EEG amplifier (according to the international 10–10 system), with the impedance of each electrode kept below 10 kΩ. For the TMS delivery, EEG signals were recorded at a sampling rate of 25 kHz with TMS-compatible amplifiers (Brainamp, Brain Products GmbH, Munich, Germany), with hardware filtering set at DC −1 kHz. Each experimental session consisted of a total of 960 pulses administered in 16 blocks of 60 pulses. The ongoing EEG activity was recorded and saved upon the conclusion of each session. The TMS was guided by a neuronavigation system (Brainsight, Rogue, Inc., Edmonton, AB, Canada) to precisely define the neuroanatomical TMS target from a T1-weighted MR image of one subject’s brain. The experimental procedure is depicted in Figure 1.

### 2.3. TMS

TMS pulses were delivered using a figure-of-eight coil connected to a magnetic stimulator (Magstim Rapid2, Magstim, Whitland, UK). The motor hotspot was defined as the position where the largest and most reliable MEPs could be obtained from the first dorsal interosseous (FDI) muscle. The RMT was determined over the left primary motor cortex (M1) targeting the FDI muscle. Surface electromyography (EMG) was recorded during the RMT assessment using disposable Ag/AgCl electrodes arranged in a belly–tendon montage over the right FDI. EMG signals were sampled at 32,768 Hz and online band-pass filtered was 10–1000 Hz. The RMT was defined as the minimum stimulation intensity required to evoke MEPs with peak-to-peak amplitudes of ≥50 μV in at least 5 out of 10 consecutive trials at rest. Across participants, the mean RMT was 83.76 ± 10.33% of maximum stimulator output (MSO) with the EEG cap. The stimulation intensity used for TMS-EEG recording was set to 90% of the individual RMT, corresponding to a mean intensity of 75.44 ± 9.28% MSO. The inter-pulse interval was set to 3–5 s. To mask the auditory click produced by the TMS, the subjects wore in-ear earplugs.

### 2.4. Phase Estimate Method

The phase at the moment of TMS cannot be reliably estimated using conventional band-pass filtering due to edge artifacts. Therefore, we applied a phase estimation method based on auto-regression (AR) forward prediction, following the approach described in previous studies [5,25]. Specifically, a pre-stimulus segment from −501 ms to −1 ms relative to the TMS pulse was extracted. Signals were spatially filtered using a Hjorth Laplacian at C3, following the discrete Laplacian/central-difference ‘source derivation’ principle proposed by Hjorth [26], computed as:

$$L_{C3}\left(t\right)=\;V_{C3}\left(t\right)-\frac{1}{4}(V_{FC1}\left(t\right)+V_{FC5}\left(t\right)+V_{CP1}\left(t\right)+V_{CP5}\left(t\right))$$

where $V_{C3}(t)$, $V_{FC1}(t)$,$\;V_{FC5}(t)$, $V_{CP1}(t)$, and $V_{CP5}\left(t\right)\;$denote the EEG potential at electrodes C3, FC1, FC5, CP1, and CP5 at time t.

The mean value and linear trend of this segment were subsequently removed. Then, for each trial, the signal was band-pass filtered using a windowed FIR filter (order: 128; passband: 8–14 Hz). An AR model (order: 30) was fitted to the pre-stimulus data, and forward prediction was applied to compensate for filter-induced distortions. Finally, the phase was extracted from the analytic signal obtained via the Hilbert transform.

To assess the accuracy of the phase estimation method used in our study, we analyzed resting-state EEG data acquired prior to TMS-EEG recordings. Five minutes of resting EEG were segmented into 1 s epochs, and the phase at the 500 ms time point was estimated using the same AR-based method. The reference phase was obtained by applying a zero-phase band-pass filter (8–14 Hz) to the same epochs and computing the phase at 500 ms using the Hilbert transform. The phase difference between the estimated and reference phases was used as a measure of estimation accuracy.

### 2.5. TMS–EEG Processing

#### 2.5.1. Preprocessing

The signals were offline preprocessed using EEGLAB and TESA [27] with custom MATLAB (R2022a) scripts. Before resampling, the 16 blocks from each participant were merged into a single continuous dataset, resampled to 1 kHz, and epoched into single trials (−1000 to 1000 ms), followed by baseline correction (−500 to −10 ms). Trials and channels showing large drifts or persistent noise were identified by visual inspection and removed. The large TMS-related electromagnetic artifact was then removed within a short window around the pulse (−2 to 10 ms) and reconstructed by interpolation. A two-step ICA strategy was adopted following the established TMS–EEG pipelines [28]. In the first ICA, components reflecting large-amplitude TMS-related artifacts were identified using TESA pop_tesa_compselect with automated rule-based detection and manual confirmation enabled, and the selected components were removed. The TMS pulse window was then removed over a wider interval (−2 to 15 ms) and reconstructed using cubic interpolation (TESA pop_tesa_interpdata, cubic with a 5 ms pre/post fitting window). The EEG data were subsequently band-pass filtered (1–100 Hz) and band-stop filtered (48–52 Hz) using a zero-phase fourth-order Butterworth filter implemented in TESA (pop_tesa_filtbutter). After filtering, the TMS pulse window (−2 to 15 ms) was removed again and recon-structed by interpolation before the second FastICA decomposition. In the second ICA, components reflecting other physiological artifacts (e.g., blink, movement, muscle, and electrode noise) were identified using the corresponding TESA rules with manual confirmation enabled and removed. Finally, the TMS pulse window (−2 to 15 ms) was re-constructed again through cubic interpolation, and the data were re-referenced to a common average.

Across participants, an average of 9.8 ± 3.1 components were removed per participant in the ICA. An average of 122.1 ± 85.0 trials (mean ± SD) were rejected as artefactual out of 960 initial trials, leaving 837.9 ± 85.0 trials for subsequent analyses. This rejection rate (~12.7% of trials) is comparable to prior TMS–EEG studies using ICA-based cleaning [29]. Based on the estimated phase at the TMS moments, the trials were divided into four groups—peak (315° to 360° and 0° to 44°), falling (45° to 134°), trough (135° to 224°), and rising (225° to 314°)—for subsequent analysis of the brain’s response characteristics when TMS was synchronized to different phases. The mean number of trials contributing to the TEP estimate in each phase group was 189.5 ± 26.7 (0°, peak), 219.8 ± 27.3 (90°, falling), 193.9 ± 27.8 (180°, trough), and 234.8 ± 33.5 (270°, rising).

#### 2.5.2. Inter-Trial Phase Coherence (ITPC) Analysis

The ITPC reflects the consistency of the neural response over experimental trials [30]. In this study, ITPC was computed using the following formula [31,32]:

$$ITPC=\frac{1}{N}\left|\sum_{k=1}^{N}e^{i\times \varphi_{k}}\right|$$

Here, N denotes the total number of epochs, and $\varphi_{k}\;$refers to the phase of the k-th trial. The phase ($\varphi_{k}$) was extracted from the analytic signal obtained via the Hilbert transform for each trial [33]. ITPC was calculated within the analysis window spanning 200 ms before to 500 ms after the TMS pulse. The resulting ITPC values fall between 0 and 1, with values approaching 1 reflecting highly consistent phase alignment across trials, whereas lower values indicate reduced inter-trial phase stability [34]. Because ITPC is a unitless, trial-number-normalized measure bounded between 0 and 1 and derived from phase angles rather than amplitude, no additional baseline normalization was applied to ITPC. In our study, we compared the ITPC values among four phase groups (peak, falling edge, trough, and rising edge) within the post-stimulation time window to assess the neural response stability under different phase conditions. A higher ITPC value indicates greater phase consistency of neural responses across trials when TMS is delivered under the same phase condition. In contrast, a lower ITPC value indicates weaker phase consistency and reduced reproducibility of neural responses across trials.

#### 2.5.3. TEP Analysis

TEPs were obtained by averaging across trials. We defined five peaks with reference to previous literature: N15 (10–20 ms), P30 (20–40 ms), N45 (30–60 ms), P60 (40–80 ms), N100 (70–150 ms), and P180 (150–230 ms) [27,35,36], where ‘N’ and ‘P’ denote negative and positive deflections, respectively. For each phase group, we calculated the peak values of each component of each subject, as well as the electroencephalogram topography at that moment point. Subsequently, to quantify the overall strength of the brain’s response to TMS, the global mean field power (GMFP) of TEPs was computed using the following formula to explore the global brain reactivity following TMS pulses [37,38]:

$$GMFP(t)=\sqrt{\left[\sum_{i}^{K}{\left(V_{i}\left(t\right)-V_{mean}\left(t\right)\right)}^{2}\right]/K}$$

where *t* is the time point, *Vi* is the voltage amplitude recorded at channel i, and K is the total number of EEG channels.

#### 2.5.4. TMS-Induced EEG Oscillation Analysis

Rhythmic neural oscillations are believed to underlie both local information processing and long-range communication across brain regions. Empirical studies using TMS–EEG have shown that when different cortical regions are perturbed, they often respond by oscillating at characteristic, intrinsic frequencies, sometimes called their natural frequencies [39,40]. These frequencies likely reflect intrinsic properties of the underlying thalamocortical architecture rather than being purely imposed by external stimulation: for example, Ferrarelli et al. demonstrated that patients with schizophrenia show a slower natural frequency in frontal thalamocortical circuits, suggesting a stable circuit-level deficit rather than just a response to the external input [41]. Theoretical models also support this idea, positing that thalamocortical loops generate ongoing, self-sustained oscillatory activity, which is then modulated (rather than driven) by incoming sensory information [42]. Therefore, TMS-induced oscillatory activity, especially its frequency and power, acts as a key indicator of the intrinsic properties and functional states of cortical circuits.

To quantify the TMS-evoked oscillatory response in the sensorimotor cortex, we computed the event-related spectral perturbation (ERSP) using a short-time Fourier transform (STFT)-based time–frequency decomposition implemented in custom MATLAB scripts. For each participant and phase condition, single-trial TMS-locked EEG epochs were transformed into the time–frequency domain, and spectral power was estimated for frequencies from 1 to 40 Hz in 0.5 Hz steps. Power estimates were then averaged across trials to obtain a mean time–frequency representation for each electrode and condition. The ERSP quantifies the log-transformed change in spectral power from the baseline period. The average value of −800 to −200 ms before TMS was used as the baseline. The corresponding baseline was subtracted from the data of each frequency band, and then division by the baseline was performed for normalization to quantify the stimulus-induced relative change in oscillatory power.

### 2.6. Statistical Analysis

All statistical analyses were performed using SPSS (version 27.0), with the significance level set at α = 0.05. The analyses were conducted separately for each metric, with mu-rhythm phase (four levels) as a within-subject repeated-measures factor. ITPC, TEPs, and TMS-evoked oscillations were analyzed at the electrode level, whereas the GMFP was analyzed as a global measure. Residual normality was assessed using the Shapiro–Wilk test. Normally distributed data were analyzed using one-way repeated-measures ANOVA, with sphericity assessed through Mauchly’s test and Greenhouse–Geisser correction applied when necessary; non-normally distributed data were analyzed using the Friedman test. Accordingly, ITPC was analyzed with repeated-measures ANOVA, whereas TEPs, GMFP, and TMS-evoked oscillations were analyzed with the Friedman test. To correct for multiple comparisons across electrodes, *p*-values for ITPC, TEPs, and TMS-evoked oscillations were adjusted using the Benjamini–Hochberg false discovery rate (FDR) procedure; this correction was not applied to the GMFP because it was not tested at the electrode level. Post hoc pairwise comparisons were performed only for significant main effects: Tukey’s HSD was used for ITPC, and Dunn’s test with Bonferroni correction was used for TEPs, TMS-evoked oscillations, and GMFP. Representative electrodes were selected from spatially contiguous significant clusters to preserve the spatial specificity of the observed effects. For the phase estimation accuracy analysis, phase angle data from the resting-state EEG were summarized using circular statistics and implemented in the CircStat toolbox for MATLAB [43].

## 3. Results

### 3.1. Phase Estimation Accuracy

**Figure 2:**
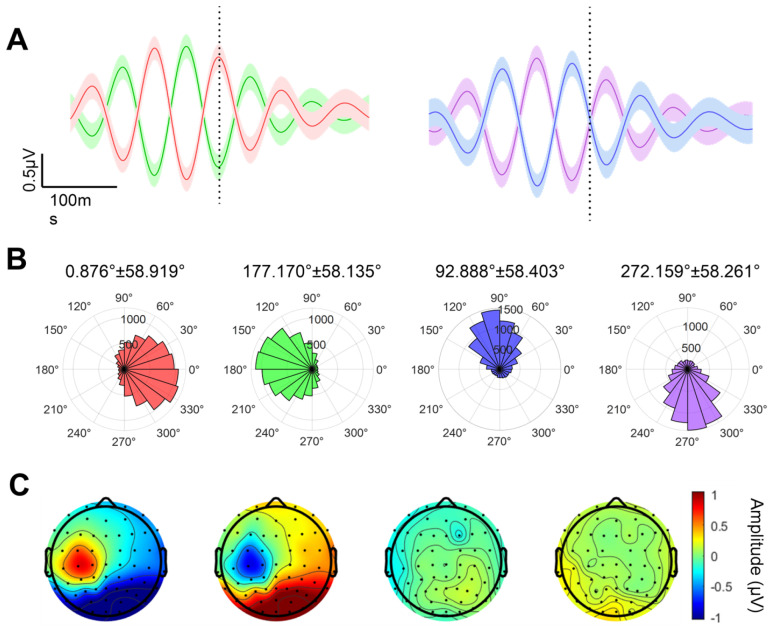
Accuracy evaluation of mu-rhythm phase estimation based on resting-state EEG. (**A**) Average of all trials across the 0°, 90°, 180°, and 270° phase groups. The black dashed line indicates the time point for phase estimation, demonstrating accurate identification of the 0°, 90°, 180°, and 270° phases. The shaded area represents ±1 standard error. In (**A**,**B**), red, green, blue, and purple represent the 0°, 90°, 180°, and 270° groups, respectively. (**B**) Histograms of the actual phase angles for the 0°, 90°, 180°, and 270° groups, showing clustering of true phases around their respective target phase values. (**C**) Topographic maps of the average amplitude of mu-rhythm oscillations (8–14 Hz) within a 3 ms time window centered at the target time point for the 0°, 90°, 180°, and 270° groups.

To assess the accuracy of the EEG mu-rhythm phase estimation method, we computed the phase error for each trial within each phase group using the resting-state EEG data collected before the subthreshold TMS–EEG experiment. The results are shown in Figure 2A,B. As observed in the time-domain signal plot in Figure 2A, the phases of the 0°, 90°, 180°, and 270° groups at the target time point all aligned with the expected phases. The actual phase values for the 0°, 90°, 180°, and 270° groups, as shown in Figure 2B, were (0.876 ± 58.919)°, (82.888 ± 58.403)°, (177.170 ± 58.135)°, and (272.159 ± 58.261)°, respectively. These phase summaries were computed using circular statistics (circular mean and circular standard deviation). V-tests revealed that the distributions of the actual angles for all four phase groups were non-uniform (*p* < 0.001), with concentrations directed toward 0°, 90°, 180°, and 270°, respectively. Finally, we generated average amplitude topographical maps of the mu-rhythm oscillations within a 3 ms time window centered at the target time point for each group, as shown in Figure 2C. These topographic maps demonstrate that the mu-rhythm oscillations we utilized originate from the left sensorimotor cortex rather than signals from other cortical regions (e.g., the occipital lobe) that share the same frequency bandwidth as the mu-rhythm.

### 3.2. ITPC

**Figure 3:**
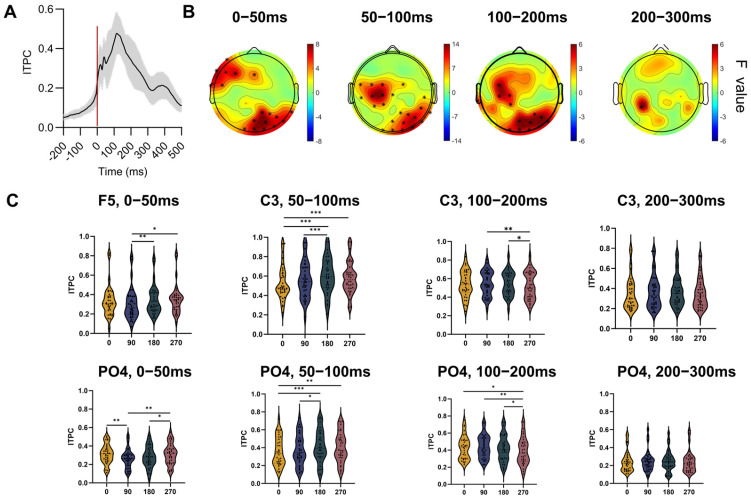
ITPC results. (**A**) Inter-trial phase clustering (ITPC) across all trials. The red solid line indicates the time of TMS. ITPC increased rapidly following TMS and subsequently decreased. (**B**) Brain regions showing significant differences in ITPC among the 0°, 90°, 180°, and 270° phase groups across time windows. The asterisk (*) indicates significant differences in ITPC among the four phase groups; * *p* < 0.05, ** *p* < 0.01, *** *p* < 0.001. (**C**) Pairwise comparisons between phase groups illustrated using representative electrodes.

The ITPC calculated for the 0°, 90°, 180°, and 270° phase groups is shown in Figure 3. Figure 3A displays the ITPC across all trials, indicating that the pre-stimulus ITPC remained below 0.2, while it increased rapidly after TMS, peaking at approximately 100 ms post-stimulation before declining. The post-stimulation period was divided into four time windows (0–50 ms, 50–100 ms, 100–200 ms, and 200–300 ms), and the average ITPC within each window was computed for the four phase groups. Statistical analyses were performed at the electrode level for each phase group, with results illustrated in Figure 3B. Significant differences in ITPC among the 0°, 90°, 180°, and 270° phase groups were observed across time windows within 200 ms post-stimulation. Specifically, during the early 0–50 ms window, significant effects emerged in the ipsilateral frontotemporal regions and the contralateral parieto-occipital regions. In the 50–100 ms and 100–200 ms windows, significant differences were identified in the ipsilateral central region and the contralateral parieto-occipital region. Representative electrodes within these significant regions were selected for post hoc pairwise comparisons between phase groups, as shown in Figure 3C.

During the 0–50 ms window at electrode F5, the ITPC was significantly lower in the 90° group than in the 180° (*p* = 0.008) and 270° (*p* = 0.011) groups. At electrode C3 during the 50–100 ms window, the ITPC of the 180° group was significantly higher than that of the 0° (*p* < 0.001) and 90° (*p* < 0.001) groups, while the ITPC of the 270° group was significantly higher than that of the 0° (*p* < 0.001) group. At C3 during the 100–200 ms window, the ITPC of the 270° group was significantly lower than that of the 90° (*p* = 0.006) and 180° (*p* = 0.010) groups.

At the contralateral posterior parietal electrode PO4 during the 0–50 ms window, the ITPC of the 0° group was significantly higher than that of the 90° (*p* = 0.002) group, and the ITPC of the 270° group was significantly higher than that of the 90° (*p* = 0.007) and 180° (*p* = 0.017) groups. During the 50–100 ms window at PO4, the ITPC of the 180° group was significantly higher than that of the 0° (*p* < 0.001) and 90° (*p* = 0.024) groups, and the ITPC of the 270° group was significantly higher than that of the 0° (*p* = 0.001) group. In the 100–200 ms window at PO4, the ITPC of the 270° group was significantly lower than that of the 0° (*p* = 0.011), 90° (*p* = 0.010), and 180° (*p* = 0.041) groups.

### 3.3. TEPs

**Figure 4:**
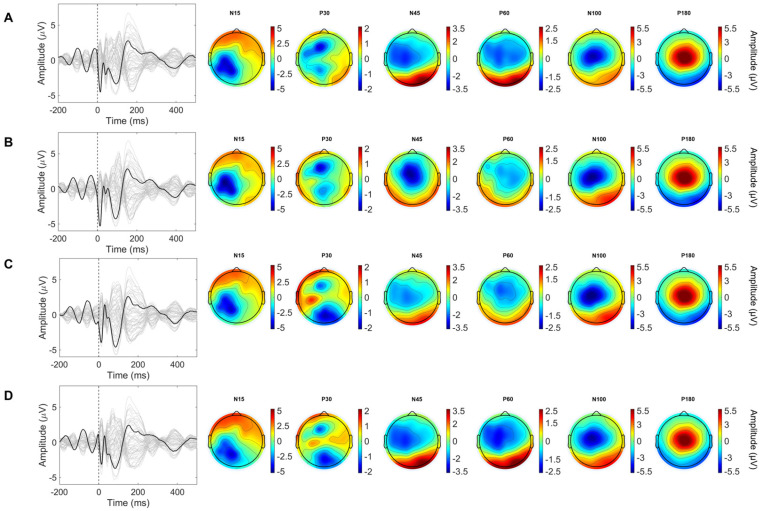
Grand-average TEP waveforms and scalp topographies across mu-phase conditions. (**A**) Grand-average TEP waveforms and topographic maps of the major components (P30, N45, P60, N100, and P180) under the 0° condition. (**B**) Grand-average TEP waveforms and topographic maps of the major components (P30, N45, P60, N100, and P180) under the 90° condition. (**C**) Grand-average TEP waveforms and topographic maps of the major components (P30, N45, P60, N100, and P180) under 180° condition. (**D**) Grand-average TEP waveforms and topographic maps of the major components (P30, N45, P60, N100, and P180) under the 270° condition.

Figure 4A–D present the grand-average TEP waveform and topographic maps of its components separately for the four phase groups. The spatiotemporal dynamics of TMS-evoked potentials over the primary motor cortex align with established literature, exhibiting six classic components: N15, P30, N45, P60, N100, and P180. P30 peaked over the left central region, N45 exhibited bilateral central distribution, P60 was maximal over the left hemisphere, while N100 and P180 were broadly distributed over bilateral frontocentral regions. Single-subject TEP waveforms at C3 for the four phase conditions are provided in Figure S1, showing the across-subject variability around the grand-average response (Supplementary Materials).

**Figure 5:**
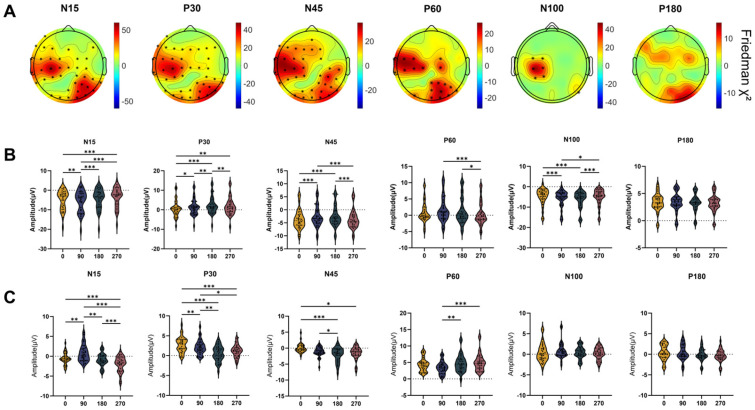
Phase-dependent differences in TEP components: scalp distribution and post hoc comparisons. (**A**) Electrode sites exhibiting significant differences across the phase groups. The black asterisks indicate electrode sites showing significant differences in TEP amplitudes across the four phase groups (Friedman test, FDR-corrected). (**B**) Post hoc pairwise comparisons between phase groups at C3 for representative components. (**C**) Post hoc pairwise comparisons between phase groups at PO4 for representative components. Asterisks indicate significant pairwise differences after multiple comparison correction. The asterisk (*) indicates significant differences in TEP components among the four phase groups, * *p* < 0.05, ** *p* < 0.01, *** *p* < 0.001.

As shown in Figure 5A, significant differences among the four phase groups were observed for N15, P30, N45, and P60 over the left central and right parieto-occipital regions, as well as for N100 over the left central area. No significant group differences were found for P180. Post hoc pairwise comparisons were performed for representative components at C3 (N15, P30, N45, P60, N100) and PO4 (N15, P30, N45, P60), with results detailed in Figure 5B,C).

At C3, the N15 amplitude at 90° was significantly higher than that at 0° (*p* = 0.008), 180° (*p* < 0.001), and 270° (*p* < 0.001), and the value at 0° was significantly higher than that at 270° (*p* < 0.001). For the P30 component, the 180° phase group exhibited a significantly higher amplitude compared to the 0° (*p* < 0.001), 90° (*p* = 0.001), and 270° (*p* = 0.006) groups, with amplitudes in the 90° and 270° groups also being significantly higher than that in the 0° group (*p* = 0.039; *p* = 0.008). For N45, amplitudes at 0° and 270° were significantly higher than those at 90° (*p* = 0.001; *p* < 0.001) and 180° (*p* = 0.001; *p* < 0.001). For P60, the amplitude at 90° was significantly higher than that at 270° (*p* = 0.039), while the amplitude at 180° was also significantly higher than that at 270° (*p* < 0.001). For N100, the amplitude at 180° was significantly higher than those at 0° (*p* < 0.001) and 270° (*p* < 0.001); the amplitude at 90° was significantly higher than that at 0° (*p* < 0.001) and 270° (*p* = 0.011).

At PO4, the N15 amplitude at 270° was significantly higher than at 0°, 90°, and 180° (all *p* < 0.001), while the amplitude at 90° was significantly higher than at 0° (*p* = 0.003) and 180° (*p* = 0.002). For P30, the amplitude at 0° was significantly higher than at 90° (*p* = 0.006), 180° (*p* < 0.001), and 270° (*p* < 0.001); the amplitude at 90° was significantly higher than at 180° (*p* = 0.004), and the amplitude at 270° was significantly higher than that at 180° (*p* = 0.040). For N45, the amplitude at 180° was significantly higher than at 0° (*p* < 0.001) and 90° (*p* = 0.039), and the amplitude at 270° was significantly higher than at 0° (*p* = 0.039). For P60, the amplitudes at 180° and 270° were significantly higher than at 90° (*p* = 0.001; *p* < 0.001).

### 3.4. GMFP

**Figure 6:**
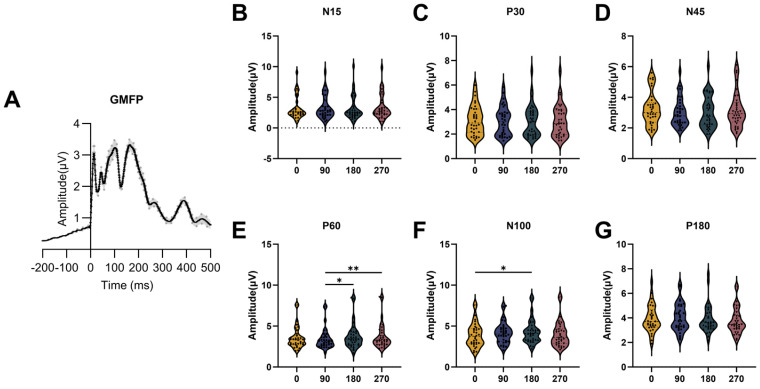
GMFP for the four phase groups. (**A**) Grand-average GMFP waveform. The N15, P30, N45, P60, N100, and P180 components are clearly identifiable. The shaded area indicates ±1 standard error (±1 SE). (**B**–**G**) Amplitudes of the N15, P30, N45, P60, N100, and P180 components for the four phase groups. The amplitudes of the early components (N15, P30, and N45) and the late component (P180) did not differ significantly among groups. In contrast, the amplitudes of the mid-latency components (P60 and N100) showed significant differences across the four phase conditions. The asterisk (*) indicates significant differences in GMFP among the four phase groups, * *p* < 0.05, ** *p* < 0.01.

The GMFP results for the four phase groups are presented in Figure 6. The P30, N45, P60, N100, and P180 components were observed in the grand-average GMFP waveform (Figure 6A). The amplitudes of the early components, N15 (F = 6.071, *p* = 0.108), P30 (F = 0.671, *p* = 0.880) and N45 (F = 4.059, *p* = 0.255), as well as the late component P180 (F = 2.859, *p* = 0.414), did not differ significantly across phase groups. In contrast, the mid-latency components, P60 (F = 14.220, *p* = 0.003) and N100 (F = 10.480, *p* = 0.015), showed clear phase-dependent modulation. For P60, the amplitude in the 90° group was significantly lower than in the 180° (*p* = 0.039) and 270° (*p* = 0.003) groups. For N100, the 180° group showed a significantly higher amplitude than the 0° group (*p* = 0.012). These findings indicate that the influence of the mu-phase on cortical reactivity is the most pronounced during the mid-latency period following stimulation. The grand-average GMFP waveforms for all four conditions are additionally shown in Figure S2 (Supplementary Materials).

### 3.5. TMS-Induced EEG Oscillations

**Figure 7:**
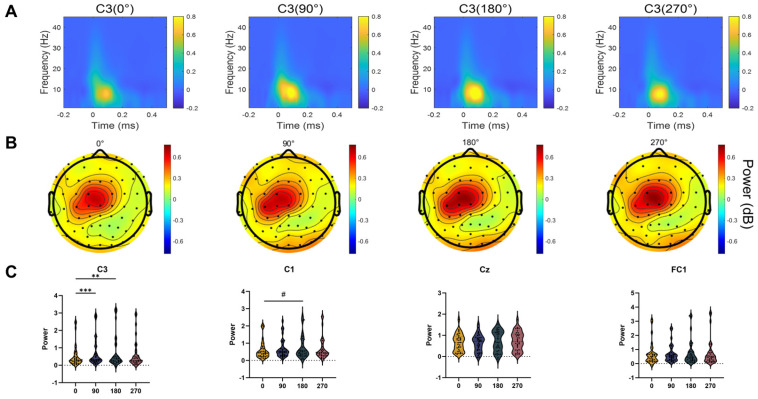
TMS-induced EEG oscillations for the four phase groups. (**A**) TMS-induced oscillatory power at electrode C3, showing an increase in the low-alpha band (7–10 Hz) within approximately 150 ms after stimulation. (**B**) Topographic maps of the ROI, showing increased low-alpha power in the left central region across all groups. (**C**) Low-alpha power at electrode C3 differed significantly among the phase groups, with post hoc comparisons indicating higher power in the 180° group than in the 0° and 90° groups. Asterisks indicate significant differences in post hoc comparisons (** *p* < 0.01, *** *p* < 0.001). The hash symbol (#) indicates a trend toward significance (*p* < 0.1).

The results of the TMS-induced EEG oscillations for the four phase groups are presented in Figure 6. We analyzed the spectral changes in the electroencephalogram (EEG) power before and after TMS pulses. A clear increase in low-alpha power (7–10 Hz) was observed in the left central region within the first 150 ms after TMS across all groups (Figure 7A,B). In the ROI analysis, the increase in low-alpha power differed significantly at electrode C3 (F = 17.960, *p* < 0.001) and showed a trend toward significance at electrode C1 (F = 7.129, *p* = 0.068). Post hoc analysis indicated that the increase at C3 in the 180° phase group was significantly higher than in the 0° (*p* = 0.006) and 90° (*p* < 0.001) groups.

## 4. Discussion

By analyzing cortical responses elicited by subthreshold TMS delivered at different phases of the mu-rhythm, we found that mu-rhythm phase modulated TMS-evoked responses, primarily in time-locked measures within 200 ms after the pulse. Specifically, phase influenced the amplitudes of several TEP components at the stimulation site, as well as ITPC across multiple post-stimulation time windows, and these effects were also evident in the parieto-occipital cortex. Moreover, the direction and magnitude of phase-related differences varied across components and time windows, indicating that ongoing mu-rhythm phase differentially shapes post-stimulation cortical responses.

We further found that the phase of the mu-rhythm primarily modulated the amplitude and ITPC of TEP components in the stimulated central region. One possible explanation is that TMS-evoked activity exhibits increased complexity, as it propagates broadly across multiple distant cortical and subcortical brain regions, which could undermine the phase-dependent modulation of TEP components in these spatially distant brain regions [44]. Interestingly, we found that within the stimulated region, the phase-related time windows differed across TEP components. Such differences are likely given that distinct TEP components are thought to reflect different neurophysiological processes. At the C3 channel, early components such as P30 and N45 have been linked to local cortical excitability dynamics; P30 correlates with corticospinal excitability measures (e.g., MEPs) [45], and N45 is modulated by ${GABA}_{A}$-mediated inhibitory processes in pharmacal studies [46]. In contrast, P60 has been linked to early afferent sensory processing related to TMS-evoked peripheral activation [28,47], whereas N100 is commonly interpreted as reflecting later inhibitory processes and ${GABA}_{B}$-mediated regulation [46,48]. We found that the amplitude of the P180 component remained unaffected by the mu-rhythm phase at the time of TMS. As a late response component, P180 reflects more distributed and potentially non-specific processes (including large-scale network-level activity and sensory contributions related to auditory and somatosensory processing evoked by the TMS pulse), which could reduce its apparent phase sensitivity [49].

Notably, P30 was the largest when TMS occurred near the mu-trough, consistent with recent phase-dependent findings that report enhanced early TEP responses at the trough [50]. This phenomenon aligns with the well-established physiological mechanism that neurons in the sensorimotor cortex exhibit a more depolarized, higher excitability state during the mu-trough-phase, resulting in significantly larger MEP amplitudes [3,9,17]. Furthermore, our analysis revealed a higher N100 amplitude under the trough condition as well, which is in line with prior evidence for mu-phase-dependent modulation of late TMS-evoked cortical responses [51], which are commonly indexed by N100-related measures. Another notable finding was that the TEP components and ITPC in the occipital cortex were modulated by the sensorimotor mu-rhythm phase, indicating that the regulatory effect of the motor cortex mu-rhythm phase on brain responses is not strictly limited to the stimulated site. This cross-regional modulation is unsurprising, given that mu-rhythm oscillations are known to play a central role in coordinating large-scale brain network functional connectivity [52,53,54], and TMS-induced neural responses are well-documented to propagate from the sensorimotor area to various cortical regions via corticocortical pathways [19,44].

Recent work has directly tested how mu-phase shapes TMS-evoked EEG responses. Using suprathreshold stimulation, Perera et al. reported mu-phase modulation of both early and late TEPs [51], with stronger late responses at the trough, and Wischnewski et al. reported phase effects on early responses in older adults and stroke survivors [50]. Notably, both studies employed suprathreshold stimulation intensities, which constitute a common limitation of such paradigms. Under these conditions, TMS-evoked brain responses are often indexed by MEPs and may therefore be influenced by non-cortical factors, including spinal excitability, peripheral sensory feedback, motor reflexes, and potential saturation effects. Collectively, these factors may mask the intrinsic cortical phase-dependent modulation of TMS-evoked responses [16]. Here, we used subthreshold TMS with TMS–EEG readouts to probe cortical responses while reducing overt corticospinal output. To date, studies using TMS–EEG to investigate phase influences on cortical responses remain relatively scarce. Ding et al. found that TMS of the motor cortex at 100% RMT intensity, timed with the occipital α-rhythm phase, could affect the spatiotemporal dynamics of the cortical response [55]. Desideri et al. applied TMS at the negative and positive peaks of the sensorimotor μ-oscillation and showed that negative peak-triggered 90% RMT TMS was associated with an enhanced N100 relative to positive peak-triggered stimulation [18]. Significant clusters were located over the sensorimotor area, in line with the present findings. However, they compared only two-phase conditions of the μ-rhythm, whereas the present study divided the μ-rhythm into four phase bins. In addition to the N100, we observed phase-dependent modulation of early- and mid-latency responses, ITPC, and GMFP. Notably, they did not detect a phase effect on TMS-induced EEG oscillations. By contrast, we found significant phase differences in low-frequency alpha oscillatory power, with greater power in the negative-peak condition than in the positive-peak and descending-phase conditions. One possible explanation is the different alpha ranges analyzed. Desideri et al. examined the 8–12 Hz alpha band, whereas we selected 7–10 Hz on the basis of the time–frequency characteristics. Indeed, when we repeated the analysis using the entire alpha band, we also found no significant phase-dependent effect. The two studies also differed in how post-TMS oscillatory activity was quantified. Desideri et al. measured induced, that is, non-time-locked, activity only. Our ERSP analysis, in contrast, captured both time-locked and non-time-locked components. This methodological difference may be another important reason for the discrepant findings.

Our study revealed that different mu-phases selectively modulated early- and mid-TEP components, GMFP, and ITPC and that they were also associated with changes in power in the 7–10 Hz range. These findings suggest that mu-phase-dependent TMS–EEG responses may provide electrophysiological markers of the cortical excitation–inhibition state and may be useful for characterizing brain states [56]. Cortical excitability measurements based on TEPs have shown promise for predicting treatment responses in clinical settings. Prior work has linked reduced/absent M1 N100 to poorer motor recovery after stroke [57] and enhanced N45 to experimentally induced pain states [58], highlighting the potential relevance of these components as biomarkers. Rather than implying immediate therapeutic efficacy, our results identify phase-related TMS-evoked features that may be relevant for future TMS–EEG studies. Recent methodological work distinguishes EEG-informed TMS, in which online EEG readouts are used to guide parameter selection before data collection, from EEG-triggered TMS, in which predefined stimulation rules are aligned with ongoing EEG features, and from closed-loop TMS, in which post-stimulus EEG readouts are used to adapt stimulation pulse by pulse toward predefined neural targets [59]. Within this framework, our findings support further evaluation of mu-rhythm phase as a candidate signal for prospective state-dependent stimulation, while recognizing that early TEP components remain difficult to separate from artifacts and noise.

A recent Registered Report suggests that probing M1 with 90% RMT may be insensitive to detecting paired associative stimulation (PAS)-induced aftereffects, indicating a potential limitation of subthreshold stimulation when tracking protocol-induced aftereffects [60]. In contrast, our study focuses on momentary state-dependent modulation by ongoing mu-rhythm phase rather than neuromodulatory plasticity. We therefore interpret our phase-related TEP differences as reflecting transient brain-state effects rather than longer lasting aftereffects. Beyond stimulation intensity, Lucarelli et al. demonstrated that the TMS pulse waveform and current direction could shape the TEP components by recruiting distinct cortico-cortical pathways [61], and subsequent work further found that these parameters modulated neural oscillations at specific frequencies in a region-dependent manner, with distinct patterns in the alpha (8–12 Hz) and beta (13–30 Hz) bands [62]. However, in the present study, we focused on the effects of the mu-phase on the TEP components and frequency-specific oscillatory dynamics. To minimize the potential con-founding effects of the stimulation parameters, we controlled these factors as strictly as possible throughout the experiment. Specifically, neuronavigation was used to ensure a stable and consistent stimulation target and coil current direction across the entire session, and stimulation was delivered with a Magstim Rapid^2^ stimulator and a figure-of-eight coil using a fixed pulse waveform. Taken together, these procedures were intended to minimize the influence of stimulation parameter variability on our findings and to ensure that the observed effects were attributable to differences in mu-rhythm phase. Notably, Lucarelli et al.’s findings that TEP features and oscillatory responses are shaped by stimulation parameters further suggest that TMS–EEG features could be used as feedback markers to optimize stimulation parameters, such as coil orientation and pulse waveform, thereby enabling the development of more effective closed-loop TMS paradigms and improving neuromodulatory outcomes. This represents a promising direction for future research.

## 5. Limitations

In addition, biological sex was not included as a primary analytical variable because previous studies suggest that TMS–EEG measures such as TEPs, ITPC, and GMFP are influenced predominantly by brain states and stimulation parameters, whereas the independent effects of sex on these cortical responses are generally weak or inconsistent [63,64]. Given the within-subject design used in this study to compare mu-rhythm phases, sex differences are unlikely to constitute a major source of variance in phase effects, and were therefore not included in the main analysis. This study has several limitations that should be considered when interpreting the findings. First, we employed an offline analysis approach to investigate the impact of the mu-rhythm phase on the TMS-evoked brain response. In the future, our findings need to be applied to high real-time closed-loop TMS systems to validate the efficacy of EEG phase-dependent stimulation strategies in real-time conditions. Second, this study used an AR model-based method for EEG phase prediction; potential phase estimation errors could have influenced the observed modulation effects. Given the current technical challenges in achieving perfectly accurate EEG phase estimation, developing more precise phase estimation methods remains an important direction for future research. Finally, although RMT was determined using EMG recordings, EMG was recorded from the FDI only and MEPs were not monitored during the TMS–EEG session. Therefore, we cannot fully exclude that occasional motor activation occurred in some participants, potentially involving adjacent intrinsic hand muscles with different thresholds. Accordingly, the stimulation intensity should be interpreted as subthreshold with respect to the FDI RMT, but not necessarily subthreshold for all hand muscles. Future studies should employ multi-muscle EMG and concurrent EMG monitoring during TMS–EEG to verify strictly subthreshold stimulation throughout the experiment.

## 6. Conclusions

This study demonstrates that the mu-rhythm phase profoundly shapes both local and large-scale cortical responses to subthreshold TMS. By using TMS–EEG rather than MEP-based measures, we revealed phase-dependent modulation patterns across multiple TEP components and cortical regions. These findings not only deepen the understanding of how intrinsic oscillatory states regulate cortical excitability but also provide empirical support for designing phase-dependent, closed-loop neuromodulation strategies. Phase-related TEP components identified here may serve as promising biomarkers for future individualized TMS interventions.

## Data Availability

The dataset is available on request from the authors.

## Supplementary Materials

The following supporting information can be downloaded at: https://www.mdpi.com/article/10.3390/bioengineering13040391/s1. Figure S1. Single-subject TEPs at C3 for the four mu-phase conditions (gray) and grand average (black). The TMS artifact removal/interpolation window (−2 to 15 ms) is shaded, and dashed lines indicate predefined latency ranges for N15, P30, N45, P60, N100, and P180. Figure S2. Global Mean Field Power (GMFP) across conditions. Grand-average GMFP (µV) time courses for the four conditions (0°, 90°, 180°, and 270°). GMFP was computed as the spatial standard deviation of the EEG potentials across all electrodes at each time point, providing a global measure of cortical response strength. The vertical line at 0 ms marks stimulus onset; the epoch spans −100 to 300 ms.
