# Supplementary material for: Mu-Rhythm Phase Modulates Cortical Reactivity to Subthreshold TMS: A TMS–EEG Study
Source: Bioengineering (Basel). 2026 Mar 27;13(4):391. doi: 10.3390/bioengineering13040391 (PMC13114207; doi:10.3390/bioengineering13040391)
Supplement: Supplementary file 1 [file bioengineering-13-00391-s001.zip › bioengineering-4160379-supplementary.pdf]

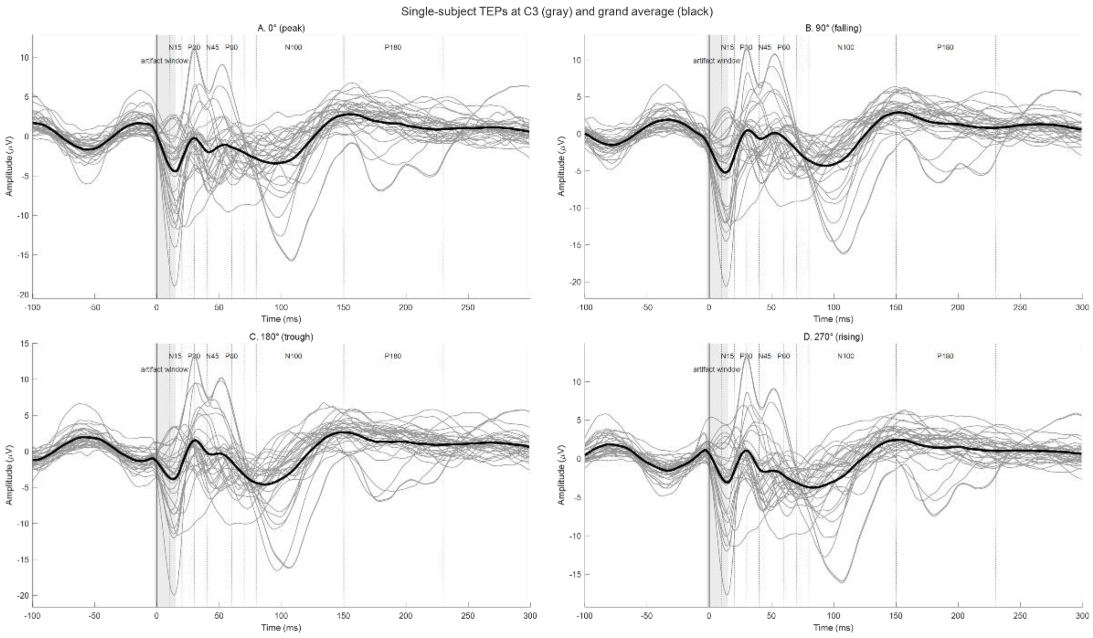

Figure S1. Single-subject TEPs at C3 for the four mu-phase conditions (gray) and grand average (black). The TMS artifact removal/interpolation window (−2 to 15 ms) is shaded, and dashed lines indicate predefined latency ranges for N15, P30, N45, P60, N100, and P180.

TMS-evoked potentials (TEPs) were visualized at channel C3 for the four mu-phase conditions (0°/90°/180°/270°) by plotting subject-level average waveforms (gray) together with the grand average (black) in the −100 to 300 ms window (Figure S1). The TMS artifact removal/interpolation window (−2 to 15 ms) is shaded to indicate the time range potentially affected by interpolation/residual artifact. Dashed lines mark the predefined latency ranges for N15, P30, N45, P60, N100, and P180 and were used to define time windows for subsequent mean-amplitude extraction.

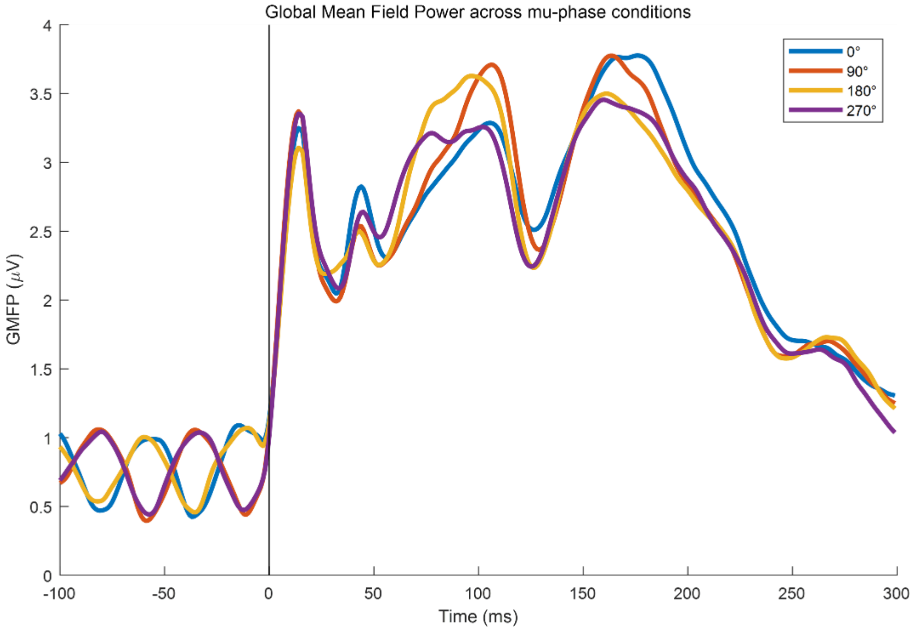

Figure S2. Global Mean Field Power (GMFP) across conditions. Grand-average GMFP (μV) time courses for the four conditions (0°, 90°, 180°, and 270°). GMFP was computed as the spatial standard deviation of the EEG potentials across all electrodes at each time point, providing a global measure of cortical response strength. The vertical line at 0 ms marks stimulus onset; the epoch spans −100 to 300 ms.

GMFP provides a reference-independent, whole-scalp summary of response magnitude and is insensitive to polarity inversions, making it suitable for comparing overall cortical reactivity across conditions. In Figure S2, the post-stimulus GMFP profiles show largely similar temporal dynamics across the four orientations, with only modest amplitude differences at selected latencies. These results complement the channel-level waveforms by confirming that the main evoked response is present in all conditions at the global level.
